# HIV Care Outcomes Among Blacks with Diagnosed HIV — United States, 2014

**DOI:** 10.15585/mmwr.mm6604a2

**Published:** 2017-02-03

**Authors:** Andre F. Dailey, Anna Satcher Johnson, Baohua Wu

**Affiliations:** 1 Division of HIV/AIDS Prevention, National Center for HIV, Viral Hepatitis, STD, and TB Prevention, CDC.

Since the release of the National HIV/AIDS Strategy (NHAS) (*1*) and the establishment of the federal Human Immunodeficiency Virus (HIV) Care Continuum Initiative (*2*), federal efforts have accelerated to improve and increase HIV testing, care, and treatment and to reduce HIV-related disparities in the United States. National HIV Surveillance System (NHSS)* data are used to monitor progress toward reaching NHAS goals,^†^ and recent data indicate that blacks have lower levels of care and viral suppression than do persons of other racial and ethnic groups (*3*). Among persons with HIV infection diagnosed through 2012 who were alive at year-end 2013, 68.1% of blacks received any HIV medical care compared with 74.4% of whites (*3*). CDC used NHSS data to describe HIV care outcomes among blacks who received a diagnosis of HIV. Among blacks with HIV infection diagnosed in 2014, 21.9% had infection classified as HIV stage 3 (acquired immunodeficiency syndrome [AIDS]) at the time of diagnosis compared with 22.5% of whites; 71.6% of blacks were linked to care within 1 month after diagnosis compared with 79.0% of whites. Among blacks with HIV infection diagnosed through 2012 who were alive on December 31, 2013, 53.5% were receiving continuous HIV medical care compared with 58.2% of whites; 48.5% of blacks achieved viral suppression compared with 62.0% of whites. Intensified efforts and implementation of effective interventions and public health strategies that increase engagement in care and viral suppression among blacks (*1*,*4*) are needed to achieve NHAS goals.

All states, the District of Columbia, and U.S. territories report cases of HIV infection and associated demographic and clinical information to NHSS. CDC analyzed data for persons aged ≥13 years reported through December 2015 from 33 jurisdictions^§^ with complete laboratory reporting.^¶^ These jurisdictions accounted for 65.3% of blacks living with diagnosed HIV infection at year-end 2013 in the United States. Stage 3 classification and linkage to care were assessed among blacks living in any of the 33 jurisdictions at the time of HIV diagnosis in 2014. A stage 3 classification was defined as having a CD4 count of <200/*µL*, CD4 percentage of total lymphocytes of <14, or documentation of an AIDS-defining condition ≤3 months after a diagnosis of HIV infection. Linkage to care was defined as having documentation of ≥1 CD4 count or percentage or viral load (VL) tests ≤1 month after HIV diagnosis. Retention in care and viral suppression were assessed among blacks with HIV diagnosed by December 31, 2012, and who were alive and resided (based on the most recent known address) in any of the 33 jurisdictions as of December 31, 2013 (i.e., persons living with diagnosed HIV). Retention in HIV care, defined as having two or more CD4 or VL tests ≥3 months apart, and viral suppression, defined as a VL of <200 copies/mL at most recent test, were assessed for 2013. Data were statistically adjusted by using multiple imputation techniques to account for missing HIV transmission categories (*5*).

In the 33 jurisdictions, 12,269 blacks received a diagnosis of HIV infection in 2014. Among these, 21.9% had infections classified as stage 3 at diagnosis (Table 1). Among males, 20.9% had a stage 3 classification, compared with 24.8% of females. The highest percentage of infections classified as stage 3 among different age groups were reported in persons aged ≥55 years (38.2%); stage 3 classifications increased with age group. By transmission category, males with infection attributed to injection drug use (IDU) had the highest percentage (32.5%) of infections classified as stage 3, followed by males with infection attributed to heterosexual contact (32.2%).

**TABLE 1:** Number and percentage of HIV infection diagnoses among blacks aged ≥13 years who were stage 3 (AIDS) at the time of diagnosis — National HIV Surveillance System, 33 jurisdictions,* United States, 2014

Characteristic	No. HIV diagnoses	Stage 3 (AIDS) at diagnosis^†^ no. (%)
**Sex**		
Male	9,121	1,908 (20.9)
Female	3,148	780 (24.8)
**Age group at diagnosis (yrs)**		
13–24	3,539	362 (10.2)
25–34	3,832	700 (18.3)
35–44	2,106	630 (29.9)
45–54	1,642	557 (33.9)
≥55	1,150	439 (38.2)
**Transmission category^§^**		
Male-to-male sexual contact	7,393	1,374 (18.6)
Injection drug use		
Male	378	123 (32.5)
Female	276	74 (26.9)
Male-to-male sexual contact and injection drug use	187	37 (19.6)
**Heterosexual contact^¶^**		
Male	1,144	369 (32.2)
Female	2,859	700 (24.5)
Other**		
Male	19	6 (31.6)
Female	14	6 (41.2)
**Total**	**12,269**	**2,688 (21.9)**

**Abbreviations:** AIDS = acquired immunodeficiency syndrome; HIV = human immunodeficiency virus.

* The 33 jurisdictions were Alabama, Alaska, California, District of Columbia, Georgia, Hawaii, Illinois, Indiana, Iowa, Louisiana, Maine, Maryland, Massachusetts, Michigan, Minnesota, Mississippi, Missouri, Nebraska, New Hampshire, New Mexico, New York, North Dakota, Oregon, South Carolina, South Dakota, Tennessee, Texas, Utah, Virginia, Washington, West Virginia, Wisconsin, and Wyoming.

^†^ Stage of disease at diagnosis of HIV infection based on first CD4 test performed or documentation of an AIDS-defining condition ≤3 months after a diagnosis of HIV infection.

^§^ Data statistically adjusted to account for missing transmission categories.

^¶^ Heterosexual contact with a person known to have or to be at high risk for HIV infection.

** Includes persons with diagnosed infection attributed to hemophilia, blood transfusion, perinatal exposure, and risk factors not reported or not identified.

Overall, 8,780 (71.6%) of the 12,269 blacks with HIV infection diagnosed during 2014 were linked to care ≤1 month after HIV diagnosis; the percentage of persons linked to care increased with increasing age group (Table 2). Overall, 70.0% of males and 76.2% of females were linked to care. By transmission category and age group, males aged 13–24 years with infection attributed to male-to-male sexual contact and IDU accounted for the lowest percentage of persons linked to care (54.9%), followed by males aged 25–34 years with infection attributed to heterosexual contact (63.0%).

**TABLE 2:** Linkage to HIV medical care within 1 month after HIV diagnosis,* among blacks aged ≥13 years, by age group and selected characteristics — National HIV Surveillance System, 33 jurisdictions,^†^ United States, 2014

Characteristic	Age group (yrs)										Total	
	13–24		25–34		35–44		45–54		≥55			
	No. HIV diagnoses	No. linked^§^ (%)	No. HIV diagnoses	No. linked^§^ (%)	No. HIV diagnoses	No. linked^§^ (%)	No. HIV diagnoses	No. linked^§^ (%)	No. HIV diagnoses	No. linked^§^ (%)	No. HIV diagnoses	No. linked^§^ (%)
**Sex**												
Male	3,044	1,945 (63.9)	3,009	2,111 (70.2)	1,338	999 (74.7)	1,036	779 (75.2)	694	548 (79.0)	**9,121**	**6,382 (70.0)**
Female	495	353 (71.3)	823	624 (75.8)	768	584 (76.0)	606	465 (76.7)	456	372 (81.6)	**3,148**	**2,398 (76.2)**
**Transmission category^¶^**												
Male-to-male sexual contact	2,847	1,821 (64.0)	2,650	1,873 (70.7)	954	714 (74.8)	638	483 (75.7)	303	234 (77.2)	**7,393**	**5,124 (69.3)**
**Injection drug use**												
Male	30	21 (70.0)	69	51 (73.9)	67	53 (79.1)	93	66 (71.0)	119	88 (73.9)	**378**	**278 (73.6)**
Female	31	22 (71.0)	57	38 (66.7)	62	45 (72.6)	71	52 (73.2)	55	45 (81.8)	**276**	**203 (73.5)**
Male-to-male sexual contact and injection drug use	51	28 (54.9)	62	43 (69.4)	33	22 (66.7)	22	16 (72.7)	19	16 (84.2)	**187**	**125 (66.7)**
Heterosexual contact**												
Male	106	67 (63.2)	227	143 (63.0)	282	209 (74.1)	281	213 (75.8)	249	208 (83.5)	**1,144**	**841 (73.5)**
Female	455	323 (71.0)	764	584 (76.4)	705	539 (76.5)	534	412 (77.2)	400	326 (81.5)	**2,859**	**2,185 (76.4)**
**Other^††^**												
Male	9	8 (88.9)	2	1 (50.0)	2	1 (50.0)	2	1 (50.0)	4	3 (75.0)	**19**	**14 (73.2)**
Female	10	7 (70.0)	2	2 (100.0)	0	0 (0.0)	0	0 (0.0)	1	1 (100.0)	**14**	**10 (76.5)**
Total	3,539	2,298 (64.9)	3,832	2,735 (71.4)	2,106	1,583 (75.2)	1,642	1,244 (75.8)	1,150	920 (80.0)	**12,269**	**8,780 (71.6)**

**Abbreviation:** HIV = human immunodeficiency virus.

* Data include persons with a diagnosis of HIV infection, regardless of stage of disease at diagnosis.

^†^ The 33 jurisdictions were Alabama, Alaska, California, District of Columbia, Georgia, Hawaii, Illinois, Indiana, Iowa, Louisiana, Maine, Maryland, Massachusetts, Michigan, Minnesota, Mississippi, Missouri, Nebraska, New Hampshire, New Mexico, New York, North Dakota, Oregon, South Carolina, South Dakota, Tennessee, Texas, Utah, Virginia, Washington, West Virginia, Wisconsin, and Wyoming.

^§^ One or more CD4 or viral load tests performed within 1 month after HIV diagnosis during 2014.

^¶^ Data statistically adjusted to account for missing transmission categories.

** Heterosexual contact with a person known to have or to be at high risk for HIV infection.

^††^ Includes persons with diagnosed infection attributed to hemophilia, blood transfusion, perinatal exposure, and risk factors not reported or not identified.

Among 257,316 blacks aged ≥13 years living with diagnosed HIV in 33 jurisdictions on December 31, 2013, approximately half (53.5%) were retained in care (Table 3), including 52.4% of males and 55.6% of females. A lower percentage of persons aged 13–34 years were retained in care (50.3%) than were persons aged ≥35 years (54.4%). By transmission category and age group, males aged 25–34 years with infection attributed to IDU accounted for the lowest percentage retained in care (38.1%), followed by males aged 13–24 years with infection attributed to heterosexual contact (39.4%). VL suppression at the most recent test was achieved by 48.5% of persons (Table 3); a higher percentage of females had suppressed VL (49.8%) than did males (47.9%). Among all age groups, the lowest level of VL suppression was among persons aged 13–24 years (39.7%); VL suppression increased with increasing age group. Females aged 13–24 years with infection attributed to IDU had the lowest level of viral suppression (29.7%), followed by males aged 13–24 years with infection attributed to heterosexual contact (31.2%).

**TABLE 3:** Retention in HIV medical care and viral suppression among blacks aged ≥13 years with HIV infection diagnosed by December 31, 2012,* who were alive on December 31, 2013, by age group and selected characteristics — National HIV Surveillance System, 33 jurisdictions,^†^ United States, 2014

Characteristic	Total no.	Retained in care in 2013**^§^**	Viral suppression^¶^
		No. (%)	No. (%)
Age ≥13 yrs**			
**Sex**			
Male	**170,740**	89,475 (52.4)	81,816 (47.9)
Female	**86,576**	48,149 (55.6)	43,095 (49.8)
**Transmission category^††^**			
Male-to-male sexual contact	**103,681**	55,110 (53.2)	50,927 (49.1)
**Injection drug use**			
Male	**27,507**	13,187 (47.9)	11,914 (43.3)
Female	**18,806**	10,315 (54.8)	8,931 (47.5)
Male-to-male sexual contact and injection drug use	**11,691**	6,697 (57.3)	5,779 (49.4)
**Heterosexual contact** ^§§^			
Male	**25,700**	13,333 (51.9)	12,359 (48.1)
Female	**65,385**	36,408 (55.7)	33,199 (50.8)
Other^¶¶^	**4,546**	2,576 (56.7)	1,803 (39.7)
**Total**	**257,316**	**137,624 (53.5)**	**124,911 (48.5)**
Age 13–24 yrs**			
**Transmission category^††^**			
Male-to-male sexual contact	**10,001**	5,059 (50.6)	4,102 (41.0)
**Injection drug use**			
Male	**127**	51 (40.2)	42 (33.1)
Female	**219**	102 (46.6)	65 (29.7)
Male-to-male sexual contact and injection drug use	**246**	120 (48.8)	96 (39.0)
**Heterosexual contact** ^§§^			
Male	**378**	149 (39.4)	118 (31.2)
Female	**2,454**	1,319 (53.7)	953 (38.8)
Other^¶¶^	**3,222**	1,884 (58.5)	1,238 (38.4)
**Total**	**16,646**	**8,684 (52.2)**	**6,614 (39.7)**
Age 25–34 yrs**			
**Transmission category^††^**			
Male-to-male sexual contact	**25,031**	12,638 (50.5)	11,110 (44.4)
**Injection drug use**			
Male	**996**	379 (38.1)	326 (32.7)
Female	**1,381**	637 (46.1)	506 (36.6)
Male-to-male sexual contact and injection drug use	**1,178**	605 (51.4)	493 (41.9)
**Heterosexual contact** ^§§^			
Male	**2,337**	1,006 (43.0)	895 (38.3)
Female	**11,754**	5,907 (50.3)	4,964 (42.2)
Other^¶¶^	**588**	299 (50.9)	218 (37.1)
**Total**	**43,265**	**21,471 (49.6)**	**18,512 (42.8)**
Age 35–44 yrs**			
**Transmission category^††^**			
Male-to-male sexual contact	**23,987**	12,680 (52.9)	11,909 (49.6)
**Injection drug use**			
Male	**3,204**	1,441 (45.0)	1,311 (40.9)
Female	**3,936**	2,016 (51.2)	1,679 (42.7)
Male-to-male sexual contact and injection drug use	**2,226**	1,220 (54.8)	1,028 (46.2)
**Heterosexual contact** ^§§^			
Male	**5,835**	2,860 (49.0)	2,637 (45.2)
Female	**20,017**	10,482 (52.4)	9,549 (47.7)
Other^¶¶^	**132**	64 (48.5)	50 (37.9)
**Total**	**59,337**	**30,763 (51.8)**	**28,162 (47.5)**
Age 45–54 yrs**			
**Transmission category^††^**			
Male-to-male sexual contact	**30,176**	16,801 (55.7)	15,967 (52.9)
**Injection drug use**			
Male	**10,168**	5,098 (50.1)	4,477 (44.0)
Female	**7,644**	4,370 (57.2)	3,720 (48.7)
Male-to-male sexual contact and injection drug use	**4,956**	3,003 (60.6)	2,584 (52.1)
**Heterosexual contact** ^§§^			
Male	**9,815**	5,361 (54.6)	4,997 (50.9)
Female	**19,644**	11,535 (58.7)	10,802 (55.0)
Other^¶¶^	**287**	157 (54.7)	139 (48.4)
**Total**	**82,688**	**46,324 (56.0)**	**42,686 (51.6)**
Age ≥55 yrs**			
**Transmission category^††^**			
Male-to-male sexual contact	**14,486**	7,933 (54.8)	7,838 (54.1)
**Injection drug use**			
Male	**13,012**	6,219 (47.8)	5,758 (44.3)
Female	**5,626**	3,190 (56.7)	2,961 (52.6)
Male-to-male sexual contact and injection drug use	**3,086**	1,749 (56.7)	1,577 (51.1)
**Heterosexual contact** ^§§^			
Male	**7,335**	3,956 (53.9)	3,713 (50.6)
Female	**11,517**	7,164 (62.2)	6,931 (60.2)
Other^¶¶^	**318**	171 (53.8)	159 (50.0)
**Total**	**55,380**	**30,382 (54.9)**	**28,937 (52.3)**

**Abbreviation:** HIV = human immunodeficiency virus.

* Data include persons with a diagnosis of HIV infection regardless of stage of disease at diagnosis. Data are based on address of residence as of December 31, 2013 (i.e., most recent known address).

^†^ The 33 jurisdictions were Alabama, Alaska, California, District of Columbia, Georgia, Hawaii, Illinois, Indiana, Iowa, Louisiana, Maine, Maryland, Massachusetts, Michigan, Minnesota, Mississippi, Missouri, Nebraska, New Hampshire, New Mexico, New York, North Dakota, Oregon, South Carolina, South Dakota, Tennessee, Texas, Utah, Virginia, Washington, West Virginia, Wisconsin, and Wyoming.

^§^ Defined as having two or more CD4 or viral load tests performed ≥3 months apart during 2013, among persons diagnosed through December 31, 2012, and alive on December 31, 2013.

^¶^ Defined as having a viral load result of ≤200 copies/mL at the most recent viral load test during 2013. The cutoff value of ≤200 copies/mL was based on the U.S. Department of Health and Human Services recommended definition of virologic failure.

** Age at year-end 2013.

^††^ Data statistically adjusted to account for missing transmission categories.

^§§^ Heterosexual contact with a person known to have or to be at high risk for HIV infection.

^¶¶^ Includes persons with diagnosed infection attributed to hemophilia, blood transfusion, perinatal exposure, and risk factors not reported or not identified.

## Discussion

In 2014, among blacks aged ≥13 years with diagnosed HIV, approximately one in five (21.9%) infections were classified as stage 3 (AIDS) at the time of diagnosis and 71.6% were linked to care within 1 month of diagnosis. Among all blacks living with diagnosed HIV at year-end 2013 in the 33 jurisdictions with complete laboratory reporting, 53.5% were retained in care and 48.5% had achieved viral suppression. These percentages are far below the NHAS 2020 goals of 85% linkage to care, 90% retention in care, and 80% VL suppression, and are also below the percentages of whites who were linked to care, retained in care and with VL suppression (79.0%, 58.2%, and 62.0%, respectively). Improving health outcomes for blacks living with HIV infection is necessary to reduce HIV in the United States. Prompt linkage to care after diagnosis allows early initiation of HIV treatment, which is associated with reduced morbidity, mortality, and transmission of HIV (*6*). Findings from CDC’s report on monitoring selected HIV prevention and care objectives indicate blacks have lower HIV linkage (71.6%) and viral suppression (48.5%) percentages than do whites (79.0% and 62.0%, respectively) (*1*).

Consistent with findings from a previous report on the continuum of HIV care among blacks with diagnosed HIV based on data from 19 jurisdictions, males had lower levels of care and viral suppression than did females, and persons aged <35 years had lower levels of viral suppression than did persons aged ≥35 years (*7*). The lowest levels of care and viral suppression among blacks with HIV in these 33 jurisdictions were among persons with infection attributed to IDU and males with infection attributed to heterosexual contact. Results of analyses by sex, and transmission category and age group should be interpreted with caution because some subpopulations have small numbers. In addition to routine testing for HIV to identify persons with unrecognized infection, interventions are needed to ensure that all persons with HIV receive optimal care; tailored strategies for black persons who inject drugs, black youths, and black males who engage in heterosexual contact might be needed to achieve improvements in care outcomes. U.S. Department of Health and Human Services treatment guidelines recommend that all adults and adolescents living with HIV in the United States be offered treatment (*2*).

The findings in this report are subject to at least two limitations. First, analyses were limited to 33 jurisdictions with complete laboratory reporting of all levels of CD4 and VL test results; these 33 jurisdictions might not be representative of all blacks living with diagnosed HIV infection in the United States. Second, comparisons of numbers and percentages by sex, and transmission category and age group should be made cautiously because subpopulations vary in size and some have small numbers.

Because blacks account for a large percentage of persons living with HIV in the United States, and to address racial/ethnic disparities in HIV care outcomes, increasing the proportion of blacks living with HIV who receive optimal HIV care is critical for achieving the goals of NHAS. Through partnerships with federal, state, and local health agencies, CDC is pursuing a high-impact prevention approach to maximize the effectiveness of current HIV prevention and care methods (*8*). CDC supports projects focused on blacks to optimize outcomes along the HIV care continuum, such as HIV testing (the first essential step for entry into the continuum of care) and projects that support linkage to, retention in, and return to care for all persons infected with HIV (*9*). Among blacks, tailored strategies for subpopulations, including persons who inject drugs and young males with infection attributed to heterosexual contact, might be needed to achieve the NHAS goal of 80% of persons living with diagnosed HIV having a suppressed viral load for all population segments.

**Summary**

**What is already known about this topic?**

Blacks living with diagnosed human immunodeficiency virus (HIV) infection have lower levels of care and viral suppression than do persons of other racial groups. National HIV/Acquired immunodeficiency syndrome (AIDS) Strategy goals include 85% linkage to care, 90% retention in care, and 80% viral load suppression by 2020.

**What is added by this report?**

In 2014, 21.9% of infections diagnosed among blacks were classified as stage 3 (AIDS) at the time of diagnosis and 71.6% of blacks with HIV diagnoses were linked to care within 1 month. Among blacks living with diagnosed HIV at year-end 2013, 53.5% were retained in care and 48.5% achieved viral suppression. The lowest levels of care and viral suppression were among persons with infection attributed to injection drug use and males with infection attributed to heterosexual contact; linkage to care and viral load suppression were lower among persons aged <35 years than persons aged ≥35 years.

**What are the implications for public health practice?**

Increasing the proportion of black persons living with HIV who are receiving care is critical for achieving the National HIV/AIDS Strategy 2020 goals to reduce new infections, improve health outcomes, and decrease health disparities. Tailored strategies for black subpopulations, including persons who inject drugs and young males with infection attributed to heterosexual contact, might be needed to achieve improvements in linkage and retention in care.

## Footnotes

* NHSS is the primary source for monitoring HIV trends in the United States. The system collects, analyzes, and disseminates information about new and existing cases of HIV infection.

† NHAS was updated in July 2015 to look forward to 2020. The NHAS goals to be accomplished by 2020 are as follows: 1) 85% of all persons with newly diagnosed HIV infection to be linked to care, 2) 90% of persons living with diagnosed HIV to be retained in care, and 3) 80% of persons living with diagnosed HIV to have a suppressed viral load.

§ The 33 jurisdictions were Alabama, Alaska, California, District of Columbia, Georgia, Hawaii, Illinois, Indiana, Iowa, Louisiana, Maine, Maryland, Massachusetts, Michigan, Minnesota, Mississippi, Missouri, Nebraska, New Hampshire, New Mexico, New York, North Dakota, Oregon, South Carolina, South Dakota, Tennessee, Texas, Utah, Virginia, Washington, West Virginia, Wisconsin, and Wyoming.

¶ The criteria for complete reporting were the following: 1) the jurisdiction’s laws or regulations required reporting of all CD4 and viral load (VL) test results to the state or local health department, 2) ≥95% of all laboratory test results were reported by laboratories that conduct HIV-related testing for each jurisdiction, and 3) the jurisdiction reported to CDC ≥95% of CD4 and VL results received since at least January 2013.
