# *Erratum:* Vol. 66, No. 4

**DOI:** 10.15585/mmwr.mm6605a6

**Published:** 2017-02-10

In the cover box “National Black HIV/AIDS Awareness Day — February 7, 2017,” on page 97, the second sentence of the second paragraph should have read, “Among blacks living with diagnosed HIV infection in 2013, 54% were **retained in** care (two or more CD4 or viral load tests ≥3 months apart) and 49% had a suppressed viral load (<200 copies/mL at most recent test) (*2*).”

